# Knowledge, attitudes and behaviors on antibiotic use and resistance among healthcare workers in Italy, 2019: investigation by a clustering method

**DOI:** 10.1186/s13756-021-01002-w

**Published:** 2021-09-10

**Authors:** Martina Barchitta, Michela Sabbatucci, Francesca Furiozzi, Stefania Iannazzo, Andrea Maugeri, Francesco Maraglino, Rosa Prato, Antonella Agodi, Annalisa Pantosti

**Affiliations:** 1 Department of Medical and Surgical Sciences and Advanced Technologies “G.F. Ingrassia”, University of Catania, Catania, Italy; 2 Department Infectious Diseases, Italian National Institute of Health, Rome, Italy; 3 Directorate General Health Prevention, Communicable Diseases and International Prophylaxis, Ministry of Health, Rome, Italy; 4 Directorate General for European and International Relations, Ministry of Health, Rome, Italy; 5 Hygiene and Public Health Service, Local Health Authority Rome 3, Rome, Italy; 6 Department of Medical and Surgical Sciences, University of Foggia, Foggia, Italy; 7 AOU Policlinico “G. Rodolico - San Marco”, Catania, Italy

**Keywords:** Antibiotic use, Antimicrobial resistance, Hand hygiene, Healthcare, Public health, Health personnel, Italy

## Abstract

**Background:**

Identifying healthcare workers (HCW) who have less awareness and knowledge on antibiotic use and resistance represents a challenge for public health, since it might help the development of novel educational and training initiatives tailored on specific subgroups of professionals. This work aims to compare knowledge, attitudes and behaviors on antibiotic use and resistance across different groups of Italian HCW.

**Methods:**

We used data from the multi-country and multi-professional survey launched by the European Centre for Disease Prevention and Control between 28 January to 4 March 2019 to assess knowledge, attitude and behaviors of HCW on antibiotics, antibiotic use and resistance. We distinguished three clusters of HCW using the Two-Step Cluster analysis, based on their personal and professional characteristics (i.e. profession, role, activity as prescriber, setting, and activity as antibiotic use advisor).

**Results:**

In general, cluster 1 consisted mostly of allied healthcare workers, while clusters 2 and 3 were made up almost completely of pharmacists and medical doctors, respectively. Interestingly, healthcare workers in cluster 3 had the highest knowledge on antibiotic use and resistance. Workers in cluster 1, instead, were those reporting the highest awareness of the importance and role of hand hygiene as an infection prevention and control measure. However, HCW in cluster 2 were those who recognized more their role of advisors on prudent antibiotic use.

**Conclusions:**

Italian HCW exhibited different knowledge, attitudes, and behaviors on antibiotic use and resistance. These findings raised the need for educational and training interventions targeting specific professional groups.

**Supplementary Information:**

The online version contains supplementary material available at 10.1186/s13756-021-01002-w.

## Introduction

Antibiotic resistance represents one of the major threats for public health worldwide. It has been estimated that every year approximately 700,000 deaths are caused by infections with antibiotic-resistant microorganisms [1, 2]. With regard to the economic burden, it has been estimated that these infections could be responsible yearly for 10 million deaths by the year 2050, having a potential annual economic cost of over USD 1 trillion by 2030 worldwide [1, 3, 4].

Although antibiotic resistance is considered a multifactorial issue, the lack of awareness on this issue itself and on the prudent use of antibiotics remain the leading causes for overuse and misuse of antibiotics and inappropriate infection prevention and control (IPC) practices [2, 5–7]. Indeed, recent studies have proposed that antibiotic resistance spread was higher in those regions where awareness and knowledge of the use of antibiotics were lower. Although previous studies have focused on the antibiotic use behavior of the general public, HCW are among the most common professional figures involved in prescribing, dispensing and/or administering antibiotics, and thus playing a key role in the management of infections [6, 8–10].

Indeed, most of the epidemiological and clinical burden of infections caused by antibiotic-resistant microorganisms is associated with healthcare across the European Union (EU) and European Economic Area (EEA) [2, 11]. However, the situation in healthcare setting varied widely, depending on the bacterial species, antibiotic class and geographical region [6–8]. This raises the need for improving awareness of antibiotic resistance and use among HCW and professionals [11]. In line, the EU-level communication plans aim to support Member States in improving public and professional understanding of antibiotic resistance, also promoting more informed clinical decision-making and prudent prescribing [8–10].

In this scenario, identifying the HCW and professional figures who have less awareness and knowledge on antibiotic resistance could be a strategy for developing novel educational and training initiatives designed to drive behavior changes on antibiotic use. In 2018, the European Centre for Disease Prevention and Control (ECDC), in the framework of the European Antibiotic Awareness Day (EAAD) initiative, launched the *Survey of health-care workers’ knowledge, attitudes and behaviors on antibiotics, antibiotic use and antibiotic resistance in the EU/EEA* [12]. This was the first multi-country and multi-professional study to assess the knowledge, attitude and behaviors of HCW on antibiotics, antibiotic use and antibiotic resistance [12]. Overall, its findings added to the evidence produced by previous reports, which described HCW knowledge about antibiotics in many countries [13–17]. The initiative was conducted using an online survey tool to evaluate capabilities, opportunities and motivations that enable prudent behavior on antibiotic use in thirty European countries [18, 19]. Specifically, this survey aimed to support the development of country-specific policy and education strategies for increasing awareness, knowledge and understanding of antibiotic resistance, also contributing to the evaluation of communication campaigns targeted to HCW operating in EU/EEA [12].

The situation of antibiotic use and resistance in Italian hospitals and regions poses a major public health threat to the country, due to high level of carbapenem-resistant *Enterobacterales* and *Acinetobacter baumannii*, and methicillin-resistant *Staphylococcus aureus* [20]. During an ECDC country visit—designed to specifically discuss and assess the Italian scenario in 2017 – several factors that have contributed negatively to this situation emerged [20]. In general, the ECDC often gained the impression of little sense of urgency, institutional support, professional leadership, and coordination of activities between and within levels [20]. Thus, we conducted a secondary analysis of the ECDC survey—focusing on data from Italian HCW—to gain a better understanding of HCW knowledge and perceptions on antibiotic use and resistance. We first applied a cluster analysis to distinguish different clusters of Italian HCW, according to their personal and professional characteristics. Next, we explored the variability across clusters in terms of knowledge and attitude of HCW on antibiotic use and resistance.

## Methods

### Survey design

The ECDC launched the survey in 2019 across thirty EU/EEA countries. The development of the survey was based on a theoretical model (COM-B) to understand the Capabilities, Opportunities and Motivations, which enable prudent Behavior on antibiotic use amongst European HCW. Survey development and study design were fully described elsewhere [2, 12]. In brief, a questionnaire was developed following a systematic literature review and a Delphi consensus process. The questionnaire, fully described in the ECDC report [12], was administered via an online survey tool between 28 January to 4 March 2019. ECDC estimated around 0.2% of the European workforce as target sample size for each healthcare profession in each country, except for nursing professionals for whom a sample of 0.1% of the workforce was calculated. A quota sampling approach was used to determine the sample size for each country and profession, according to EU healthcare personnel statistics. Based on these calculations, ECDC calculated the target survey sample size for Italy as 1,285 responses overall. Specifically, the survey was developed to be administered to 479 medical doctors (both physicians and surgeons), to 354 nurses and midwives, to 140 pharmacists (both community and hospital pharmacists), to 97 dentists, and to 215 other HCW (i.e. hospital managers, pharmacy technicians, physiotherapists, biomedical scientists, and allied health professionals). The online survey was distributed by members of the Project Advisory Group and promoted via social media. Thus, although it was not possible to estimate the overall response rate, approximately 80% of EU/EEA countries achieved or exceeded their quota sample size. Participation was on a voluntary basis and data were collected anonymously and held securely in line with the General Data Protection Regulation (EU) 2016/679. All respondents participated strictly in their professional capacity, and were provided with informed consent prior to participation, according to the Declaration of Helsinki. In the current study, we aimed at analyzing data from Italian HCW who provided a complete assessment of personal and professional characteristics, used to distinguish different clusters of professionals.

### Statistical analysis

We employed a clustering approach to distinguish different groups of HCW, not only based on their profession but also considering other personal and professional characteristics. A Two-Step Cluster analysis was applied to partition the original dataset into different clusters of participants, with high within-cluster homogeneity and between-cluster variability. This algorithm, based on the Schwarz’s Bayesian Information Criterion (BIC), allowed to handle categorical and continuous variables and to choose the exact number of clusters across different clustering solutions [21]. We chose participants’ characteristics to be included into the Two-Step Clustering algorithm among the following: age, sex, profession, years in profession, role, setting, activity as prescriber and/or antibiotic use advisor, social media users. Specifically, we selected the top-five predictors (i.e. profession, role, activity as prescriber, setting, and activity as antibiotic use advisor). The number of clusters was chosen according to BIC and silhouette values. We next evaluated between-cluster differences in HCW knowledge and attitudes on antibiotic use and resistance using the Chi-Square or the Kruskal–Wallis tests. Descriptive statistics were reported as percentages or median and interquartile range (IQR). All the analyses were performed on the SPSS software (version 23.0, SPSS, Chicago, IL, USA), with significance level α of 0.05.

## Results

The European HCW completed 18,365 questionnaires. Among them, the Italian health professionals provided responses with 2167 questionnaires, exceeding (169%) the calculated target survey sample size for our country.

We analyzed data from 1693 Italian HCW who provided a complete assessment of personal and professional characteristics. In particular, the assessment was completed by 832 medical doctors, 555 pharmacists or pharmaceutical technicians, 241 allied HCW (including 85.1% of nurses), and 65 other HCW.

The Two-Step Cluster analysis distinguished three different clusters of participants, which were related strictly to the type of profession and to the activities as prescriber and/or as antibiotic use advisor. Indeed, clusters 2 and 3 were made up almost completely of pharmacists and medical doctors (99.8% and 99.7%, respectively): the former were also mostly antibiotic use advisors; the latter were both antibiotic use advisors and prescribers. Cluster 1, instead, represented a heterogeneous group with a majority of allied HCW (53.8%). Table 1 shows in detail these differences across clusters, but also the comparison of other professional and personal characteristics.

**Table 1:** Characteristics of clusters of healthcare workers (HCW) participating in the survey on antibiotic use and resistance, Italy, 2019

HCW characteristics (%)	Cluster 1 (n = 442)	Cluster 2 (n = 503)	Cluster 3 (n = 748)	p-value
Sex (female)	73.4	67.8	40.2	< 0.001
Age				< 0.001
18–45 years	42.1	59.8	30.5	
46 years-over	57.9	40.2	69.5	
Profession				< 0.001
Medical Doctor	19.5	–	99.7	
Pharmacist	12.0	99.8	0.1	
Allied HCW	53.8	0.2	0.1	
Other	14.7	–	0.1	
Years in profession				< 0.001
≤ 25 years	66.3	76.3	51.3	
> 25 years	33.7	23.7	48.7	
Role				< 0.001
Research	14.5	0.8	2.5	
Generalist	20.6	85.7	52.5	
Public Health and Management	31.7	12.5	5.3	
Specialist infection	10.9	–	6.6	
Specialist non-infection	22.4	1.0	33.0	
Setting				< 0.001
Community	4.4	–	45.9	
Governmental Organization	10.0	0.2	4.8	
Hospital	75.2	–	44.2	
Other	1.9	–	2.3	
Pharmacy	–	99.8	–	
University	8.6	–	2.8	
Prescriber (Yes)	0.7	1.8	100	< 0.001
Advice on antibiotics (Yes)	59.0	93.2	98.5	< 0.001
Use of Social Media (Yes)	60.6	64.2	56.6	0.024

*Results are reported as percentage and compared using the Chi-square test

We also noted some differences across clusters in terms of social media used, with cluster 3 showing the lowest proportion of users compared to other clusters. Moreover, we observed that the use of different platforms varied across clusters (See Additional file 1: Fig. S1). Participants in cluster 2 reported that they used mostly Facebook and Instagram while those in cluster 1 and 3 preferred to use Twitter and LinkedIn. No differences in terms of use of Google or YouTube across clusters were evident.

**Fig. 1:**
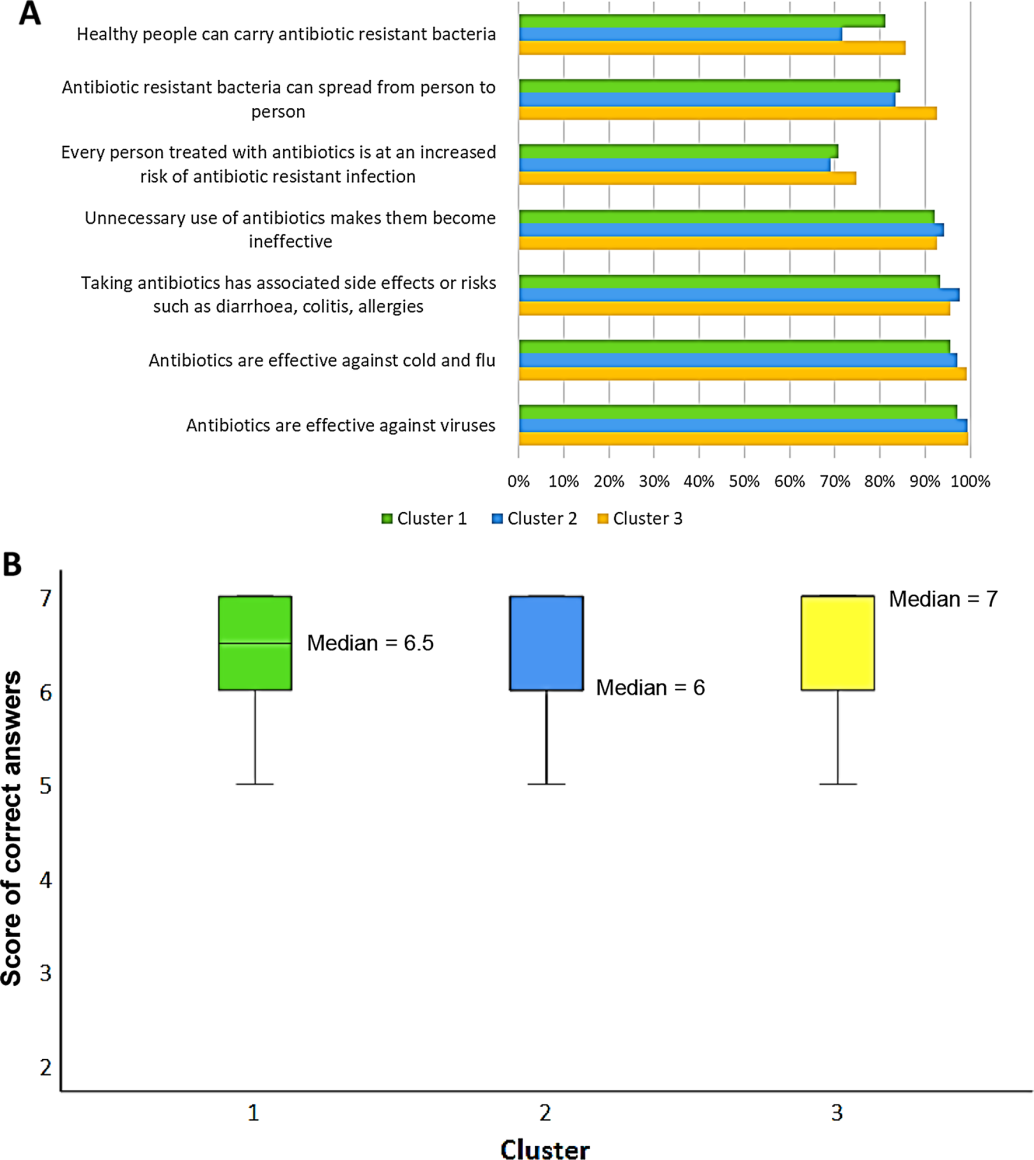
Proportion of healthcare workers stratified by cluster and correct knowledge of antibiotic use and resistance (**A**), or score (**B**). Results are reported as percentages of correct answers (**A**) or as score given by the sum of correct answers (**B**)

Next, based on the COM-B behavioral model, we analyzed the survey responses assessing the Capabilities, Opportunity, Motivation, and Behavior on antibiotic use and resistance. Specifically, participants in cluster 3 had the highest proportion of correct answers about their knowledge of the correct use of antibiotics and of antibiotic resistance in human health (“Antibiotics are effective against viruses” 99.6%; “Antibiotics are effective against cold and flu” 99.2%; “Healthy people can carry antibiotic resistant bacteria” 92.6% and “Antibiotic resistant bacteria can spread from person to person” 85.7%). In contrast, participants in cluster 2 provided the highest proportion of correct answer compared to the other participants for the question “Unnecessary use of antibiotics makes them become ineffective” (94.2%) (Fig. 1A). Using a score given by the sum of correct answers, participants belonging to cluster 3 achieved a higher value (median = 7.0, IQR = 1.0) than those in clusters 1 (median = 6.5, IQR = 1.0) and 2 (median = 6.0, IQR = 1.0) (Fig. 1B).

**Fig. 2:**
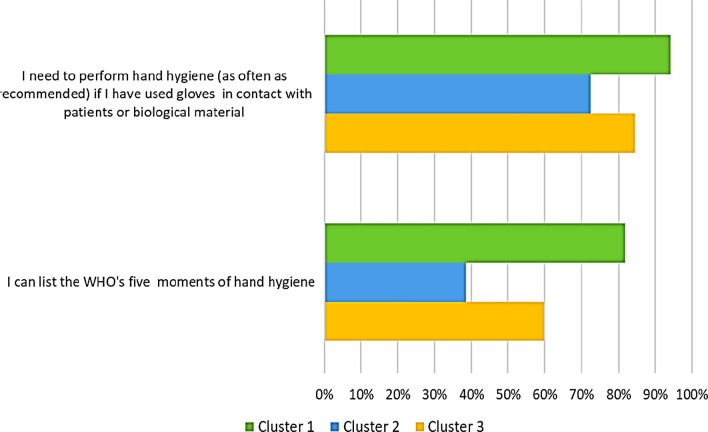
Proportion of healthcare workers stratified by cluster and competence of hand hygiene. Results are reported as percentages of participants who “agreed that they need to perform hand hygiene as often as recommended” and who “could list the WHO’s five moments of hand hygiene”

Figure 2 compares across clusters the knowledge and competence regarding hand hygiene and its critical role for the prevention and control of antibiotic resistance. Notably, the percentages of participants who reported that they “could list the WHO’s five moments of hand hygiene” and who “agreed that they need to perform hand hygiene as often as recommended” were the highest in cluster 1 (81.9% and 94.1% respectively).

**Fig. 3:**
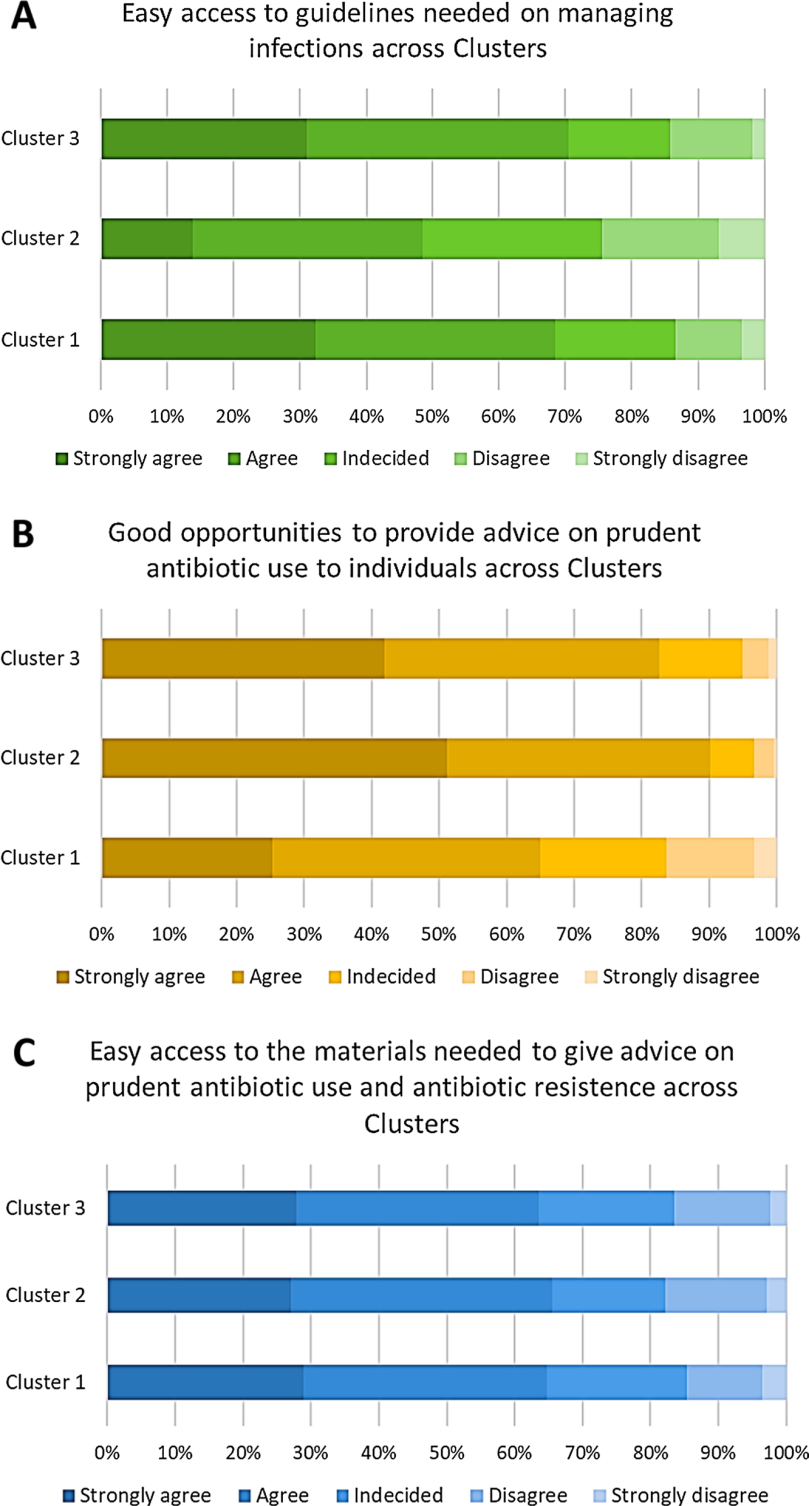
Proportion of healthcare workers stratified by cluster and availability of guidelines on managing infections (**A**) or opportunity to advice on antibiotic use (**B**) or availability of informational material (**C**). Results are reported as percentage for each degree of agreement to the indicated statement

Next, we assessed the availability of informational material and opportunities relevant for preventing and controlling antibiotic resistance for survey respondents. In particular, the percentage of participants who agreed or strongly agreed that they had easy access to guidelines on managing infections was higher in clusters 3 (70.5%) and 1 (68.5%) than in cluster 2 (48.5%) (Fig. 3A). Conversely, the percentage of participants who agreed or strongly agreed that they had good opportunities to advise individuals on prudent antibiotic use was higher in clusters 2 (90.2%) and 3 (82.6%) than in cluster 1 (64.9%) (Fig. 3B). Overall, we observed that the distribution across clusters in terms of easy access to materials for advising on prudent antibiotic use and antibiotic resistance was similar across the clusters (Fig. 3C).

**Fig. 4:**
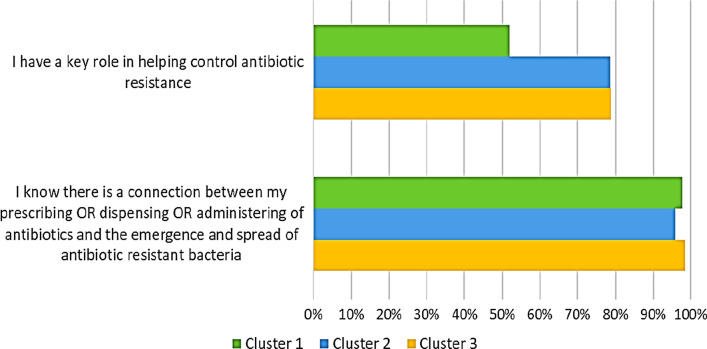
Proportion of healthcare workers stratified by cluster and awareness of their own role. Results are reported as percentage of participant who stated “I have a key role in helping control antibiotic resistance” and “I know there is a connection between prescribing or dispensing or administering of antibiotics and spread of antibiotic resistant bacteria”

As reported in Fig. 4, the proportion of respondents who recognized their role in helping control antibiotic resistance was higher in clusters 3 (78.8%) and 2 (78.6%) than in cluster 1 (52.0%). However, the proportion of respondents who strongly agreed or agreed that there is a connection between their prescribing/dispensing/administering of drugs and the emergence and spread of antibiotic-resistant bacteria and that they have a key role in helping control antibiotic resistance was higher in clusters 3 (98.4%) and 1 (97.8%) than in cluster 2 (95.8%).

**Fig. 5:**
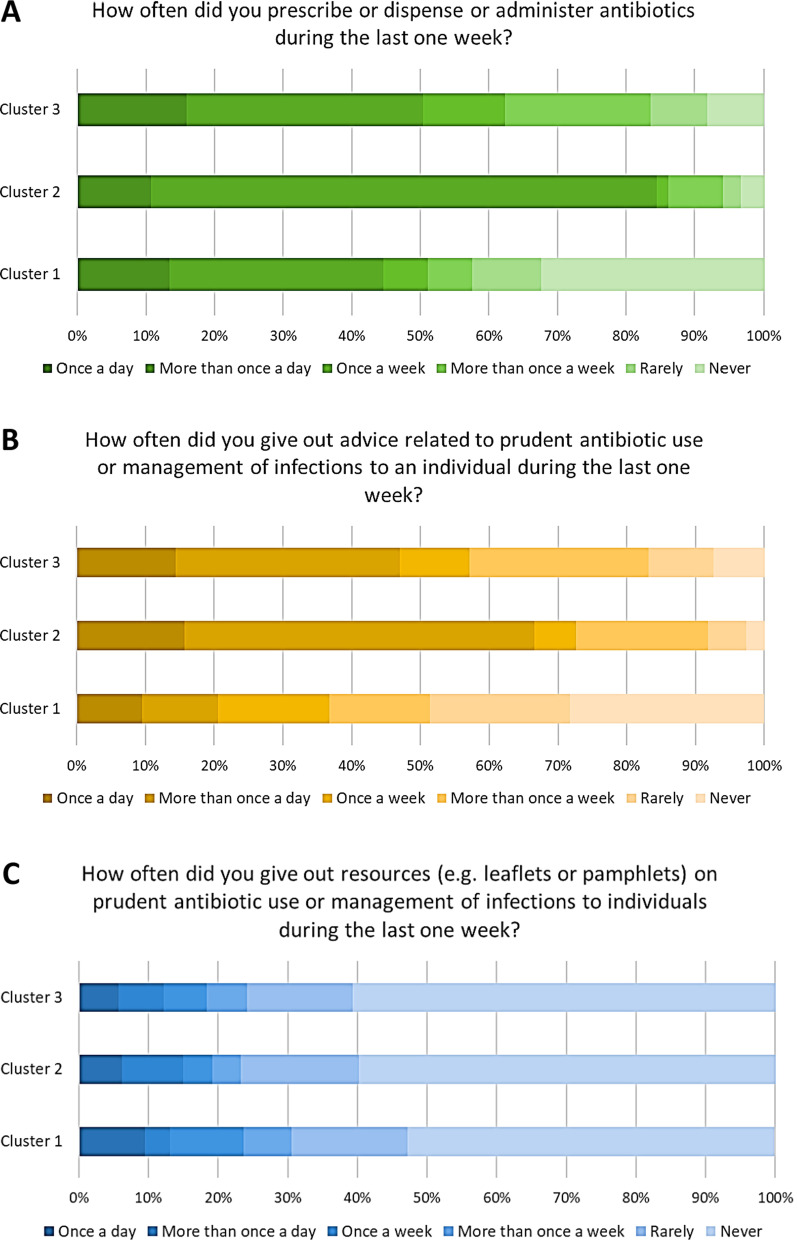
Proportion of healthcare workers stratified by cluster and frequency of prescribing/dispensing/administering antibiotics (**A**), or giving out advice (**B**) or informational resources (**C**) on antibiotic use and infection management during the last week. Results are reported as percentage of responders to the indicated questions and answers

Then, we explored the various behaviors across clusters regarding antibiotic prescription or administration and the provision of resources and advice related to the prudent use of antibiotics and infection management. The majority of participants in cluster 2 and 3 reported they had prescribed, administered or dispensed antibiotics (Fig. 5A) and had provided advice on managing infections or on the prudent use of antibiotics (Fig. 5B) more than once a week, during the last one. By contrast, most of the participants in cluster 1 reported they had prescribed, administered or dispensed antibiotics (Fig. 5A) and had given out advice on managing infections or the prudent use of antibiotics (Fig. 5B) never or rarely during the last week. The proportion of participants who reported they had never provided resources (e.g. leaflets or pamphlets) on prudent antibiotic use or management of infections in the previous week was higher (60.8%) in cluster 3 than in cluster 1 (52.7%) and 2 (59.9%; p < 0.01) (Fig. 5C).

Finally, participants in cluster 1 were more likely aware of the national action plan on antibiotic resistance and of the international antibiotic awareness campaigns EAAD and WAAW (the World Antimicrobial Awareness Week), than those in cluster 2 and 3 (p < 0.001) (see Additional file 2: Fig. S2).

## Discussion

In this study, we first distinguished three clusters of HCW among the 1,693 Italian professionals participating in the ECDC survey [12], and then compared their knowledge, attitudes and behaviors on antibiotic use and resistance. The clustering solution reached was driven mainly by differences in the type of profession and activity as prescriber and antibiotic use advisor. Indeed, for example, cluster 3 was made up largely of medical doctors who had a role of prescribers and advisors. In line with the ECDC survey [12], they were also those with the highest knowledge on antibiotic use and resistance. This was probably due to their greater awareness of the relationship between antibiotic use and resistance and their easiest access to guidelines on infection management, reasons that also emerged from the ECDC survey [12]. However, it is also worth mentioning that many studies reported an insufficient knowledge on antibiotic use among medical doctors, even in those contexts where educational training has been promoted [17]. Indeed, HCW as a whole often reported a lack of awareness of national and international initiatives to promote appropriate antibiotic use and contrast antibiotic resistance [22]. In general, our study documented scarce awareness among HCW, in line with what reported in the ECDC survey [12]. The level of awareness was higher in cluster 1, which represented a mixed group of HCW including nurses, technicians, non-prescribing medical doctors, some pharmacists, and other HCW. Participants in cluster 1 were the most aware about the national action plan on antibiotic resistance and the international antibiotic awareness campaigns EAAD and WAAW. Moreover, they were also those who most were able to recall the WHO’s five moments of hand hygiene, one of the main IPC measures against antibiotic resistance spread [23]. On the other hand, however, they reported the lowest knowledge on antibiotic use and resistance, raising the need for specific educational activities on these issues for the components of Cluster 1.

As highlighted in many reports [24–26], also pharmacists play a crucial role in ensuring the proper knowledge about antibiotic use and resistance. In our study, pharmacists were mostly included in cluster 2 and were the HCW who acknowledged most their role in the fight against antibiotic resistance. This was important, as also highlighted by the ECDC survey [12], because pharmacists and medical doctors are the key actors who prescribe antibiotics and advise individuals on the appropriate use. However, previous studies identified gaps of knowledge among community pharmacists [27, 28]. For these reasons, the introduction of specialist antibiotic pharmacists—HCW with a role in monitoring antibiotic use, advising clinicians, and educating the community—should be encouraged [29].

Our study requires certain considerations about its methodological approach and results achieved. Although we employed an approach for grouping Italian HCW according to a set of personal and professional characteristics, the resulting clustering solution mainly reflected differences in type of profession and activities as prescriber and advisor on antibiotics. Moreover, while two clusters were clearly defined (i.e., clusters 2 and 3), the third one was extremely heterogeneous for personal and professional characteristics. This did not allow us to understand whether the observed differences with the other two clusters were related to the entire cluster or to specific subgroups of HCW. Indeed, previous studies conducted in different countries suggested that knowledge and attitudes on antibiotic use and resistance might differ among HCW, and that this difference could be attributed to their level of education, professional experience, and type of hospital where they work [14, 15, 17, 30]. For instance, the study by Bai and colleagues demonstrated that in China doctors working in tertiary hospitals, who received training, and who habitually prescribed antibiotics had more knowledge on antibiotic use than their counterparts working in secondary hospitals or primary healthcare facilities [17]. Some of these factors might also explain differences in knowledge observed between studies conducted in different populations and countries [14, 15, 17, 30]. Therefore, cluster analysis could be a suitable multivariate approach to discriminate specific group of professionals who need tailored trainings and initiatives. Thus, further studies should be encouraged, also exploiting data from the ECDC survey. On the other hand, our study provided a clear description of the knowledge, attitudes and behaviors on antibiotic use and resistance among Italian HCW. In our opinion, this information will be important to contribute to the design of education and communication campaigns tailored to HCW in the framework of the Italian National Plan to fight antibiotic resistance.

## Conclusions

In conclusion, our study highlights different knowledge, attitudes, and behaviors on antibiotic use and resistance among Italian HCW. Medical doctors were those with the highest level of knowledge, probably for their greater awareness of the relationship between antibiotic use and resistance and their easiest access to guidelines. Although our analysis provides data and information that will be useful to plan future campaigns and initiatives targeting HCW, there is still a need for further studies to explore what other social and professional factors affect the level of knowledge and awareness on these issues.

## Supplementary Information

**Additional file 1: Figure S1** Proportion of healthcare workers participating in the survey on antibiotic use and antibiotic resistance, stratified by cluster and social media use, Italy, 2019 (n = 1,693). ** p-values <0.01 or *** p-values <0.001. Differential use of social media by the HCWs who participated in the ECDC survey on antibiotic use and antibiotic resistance.

**Additional file 2: Figure S2** Proportion of healthcare workers participating in the survey on antibiotic use and antibiotic resistance, stratified by cluster and awareness of the Italian national Action Plan on antibiotic resistance, the European Awareness Antibiotic Day (EAAD) and Week (WAAW), Italy, 2019 (n = 1,693). *** p-values <0.001. Differential awareness of EAAD, WAAW and the national action plan on antibiotic resistance by the HCWs who participated in the ECDC survey on antibiotic use and antibiotic resistance.

## Data Availability

The data that support the findings of this study are available from the authors upon reasonable request and with permission of ECDC.

## Abbreviations

BIC: Bayesian Information Criterion
COM-B: Capabilities, Opportunities, Motivations, Behavior
EAAD: European Antibiotic Awareness Day
ECDC: European Centre for Disease Prevention and Control
EEA: European Economic Area
EU: European Union
HCW: Healthcare workers
IPC: Infection prevention and control
SPSS: Statistical Package for Social Science
WAAW: World Antimicrobial Awareness Week
