# Supplementary material for: Knowledge, attitudes and behaviors on antibiotic use and resistance among healthcare workers in Italy, 2019: investigation by a clustering method
Source: Antimicrob Resist Infect Control. 2021 Sep 10;10:134. doi: 10.1186/s13756-021-01002-w (PMC8431867; doi:10.1186/s13756-021-01002-w)

Additional file 1

Supplementary Figure 1. Proportion of healthcare workers participating in the survey on antibiotic use and antibiotic resistance, stratified by cluster and social media use, Italy, 2019 (n = 1,693). ** p-values <0.01 or *** p-values <0.001. Differential use of social media by the HCWs who participated in the ECDC survey on antibiotic use and antibiotic resistance.

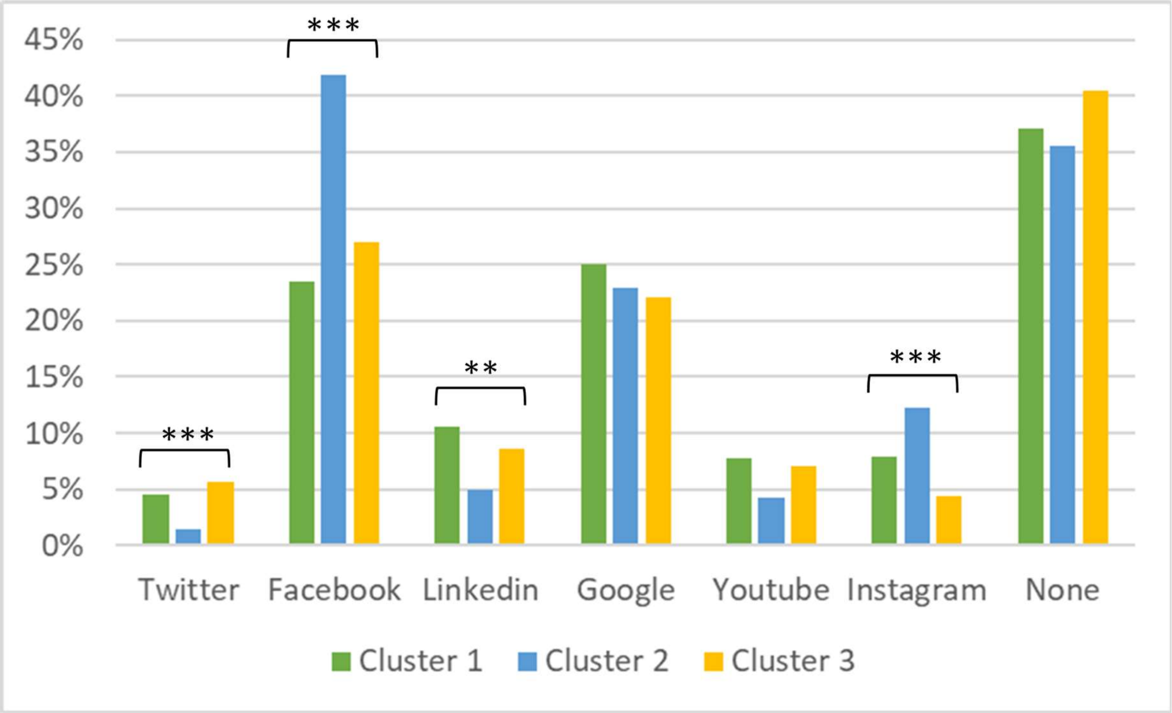

Supplement: Supplementary file 1 — Additional file 1: Figure S1 Proportion of healthcare workers participating in the survey on antibiotic use and antibiotic resistance, stratified by cluster and social media use, Italy, 2019 (n = 1,693). ** p-values <0.01 or *** p-values <0.001. Differential use of social media by the HCWs who participated in the ECDC survey on antibiotic use and antibiotic resistance. [file 13756_2021_1002_MOESM1_ESM.pdf]
