# Supplementary material for: Knowledge, attitudes and behaviors on antibiotic use and resistance among healthcare workers in Italy, 2019: investigation by a clustering method
Source: Antimicrob Resist Infect Control. 2021 Sep 10;10:134. doi: 10.1186/s13756-021-01002-w (PMC8431867; doi:10.1186/s13756-021-01002-w)

Additional file 2

Supplementary Figure 2. Proportion of healthcare workers participating in the survey on antibiotic use and antibiotic resistance, stratified by cluster and awareness of the Italian national Action Plan on antibiotic resistance, the European Awareness Antibiotic Day (EAAD) and Week (WAAW), Italy, 2019 (n = 1,693). *** p-values <0.001. Differential awareness of EAAD, WAAW and the national action plan on antibiotic resistance by the HCWs who participated in the ECDC survey on antibiotic use and antibiotic resistance.

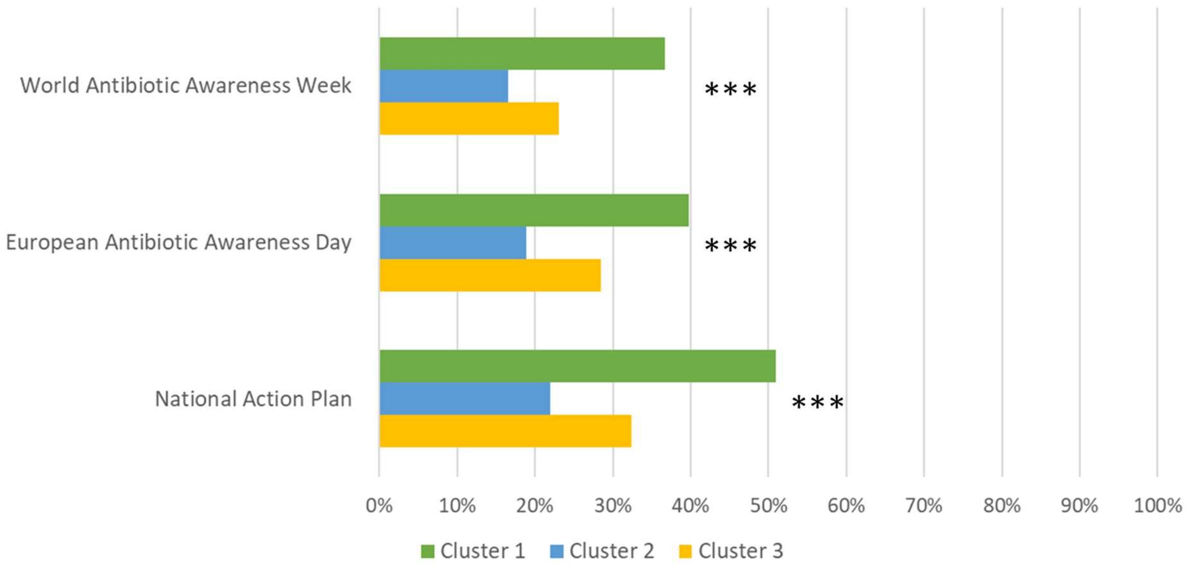

Supplement: Supplementary file 2 — Additional file 2: Figure S2 Proportion of healthcare workers participating in the survey on antibiotic use and antibiotic resistance, stratified by cluster and awareness of the Italian national Action Plan on antibiotic resistance, the European Awareness Antibiotic Day (EAAD) and Week (WAAW), Italy, 2019 (n = 1,693). *** p-values <0.001. Differential awareness of EAAD, WAAW and the national action plan on antibiotic resistance by the HCWs who participated in the ECDC survey on antibiotic use and antibiotic resistance. [file 13756_2021_1002_MOESM2_ESM.pdf]
